# Gene model for the ortholog of *gig* in *Drosophila mojavensis*

**DOI:** 10.17912/micropub.biology.001027

**Published:** 2025-08-04

**Authors:** Madeline L. Gruys, Madison A. Sharp, Corinne Croslyn, Meghan Yap-Chiongco, Laura K. Reed, Joyce Stamm, Chinmay P. Rele

**Affiliations:** 1 The University of Alabama, Tuscaloosa, AL USA; 2 University of Evansville, Evansville, Indiana, United States

## Abstract

Gene model for the ortholog of gigas ( *gig* ) in the May 2011 (Agencourt dmoj_caf1/DmojCAF1) Genome Assembly (GenBank Accession: GCA_000005175.1 ) of *Drosophila mojavensis* . This ortholog was characterized as part of a developing dataset to study the evolution of the Insulin/insulin-like growth factor signaling pathway (IIS) across the genus *Drosophila* using the Genomics Education Partnership gene annotation protocol for Course-based Undergraduate Research Experiences.

**Figure 1. Genomic neighborhood and gene model for gig in Drosophila mojavensis:**
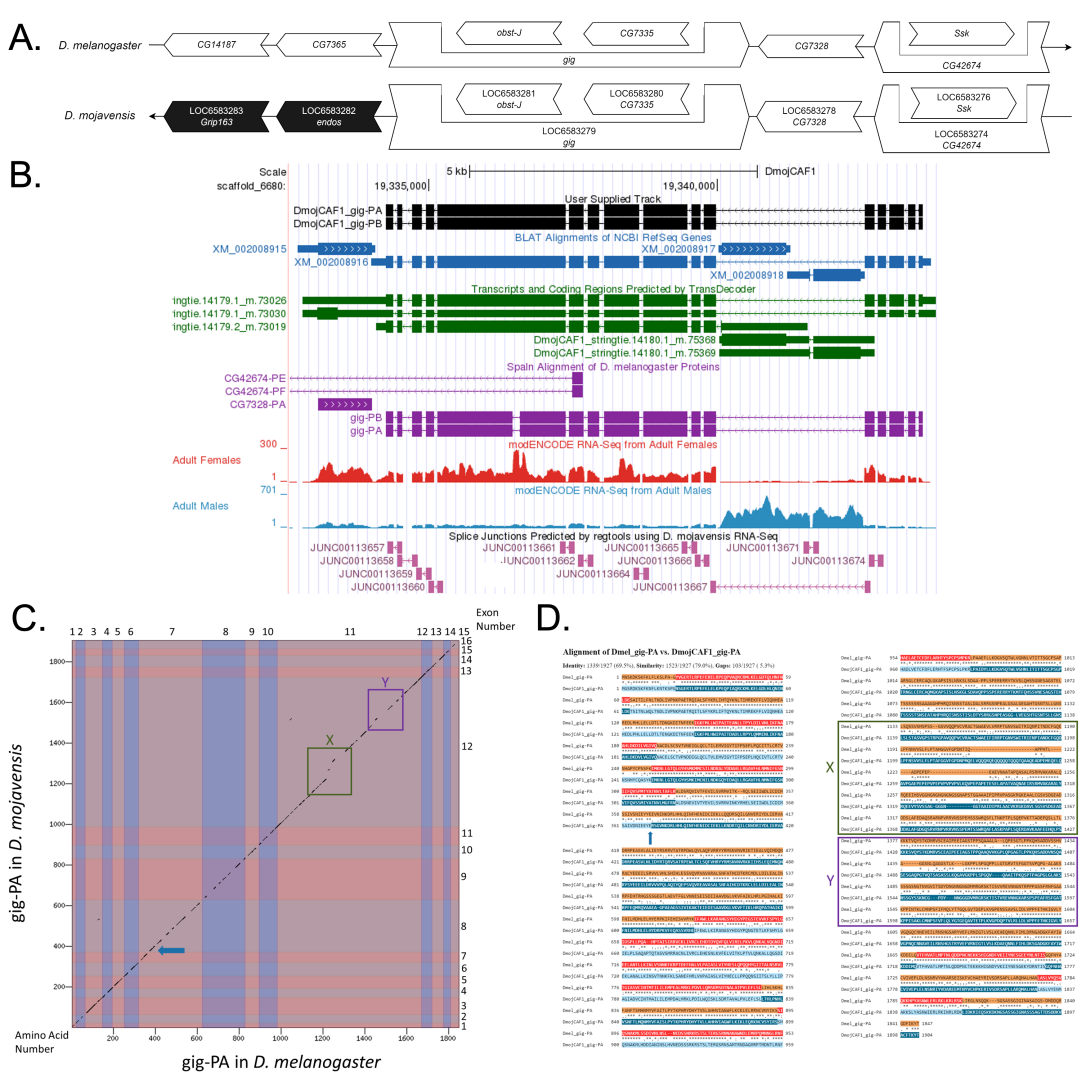
**
(A) Synteny comparison of the genomic neighborhoods for *gig* in *Drosophila melanogaster* and *D. mojavensis* .** Thin underlying arrows indicate the DNA strand within which the target gene– *gig* –is located in *D. melanogaster* (top) and *D. mojavensis* (bottom). The thin arrow pointing to the right indicates that *gig* is on the positive (+) strand in *D. melanogaster* , and the thin arrow pointing to the left indicates that *gig* is on the negative (-) strand in *D. mojavensis* . The wide gene arrows pointing in the same direction as *gig* are on the same strand relative to the thin underlying arrows, while wide gene arrows pointing in the opposite direction of *gig* are on the opposite strand relative to the thin underlying arrows. White gene arrows in *D. mojavensis* indicate orthology to the corresponding gene in *D. melanogaster* , while black gene arrows indicate non-orthology. Gene symbols given in the *D. mojavensis* gene arrows indicate the orthologous gene in *D. melanogaster* , while the locus identifiers are specific to *D. mojavensis* . **(B) Gene Model in GEP UCSC Track Data Hub (Raney et al., 2014).** The coding-regions of *gig* in *D. mojavensis* are displayed in the User Supplied Track (black); coding CDSs are depicted by thick rectangles and introns by thin lines with arrows indicating the direction of transcription. Subsequent evidence tracks include BLAT Alignments of NCBI RefSeq Genes (dark blue, alignment of Ref-Seq genes for *D. mojavensis* ), Spaln of *D. melanogaster* Proteins (purple, alignment of Ref-Seq proteins from *D. melanogaster* ), Transcripts and Coding Regions Predicted by TransDecoder (dark green), RNA-Seq from Adult Females and Adult Males (red and light blue, respectively; alignment of Illumina RNA-Seq reads from *D. mojavensis* ), and Splice Junctions Predicted by regtools using *D. mojavensis* RNA-Seq (SRP006203). Splice junctions shown have a read-depth of 100-499 supporting reads in pink. **(C) Dot Plot of gig-PA in *D. melanogaster* ( *x* -axis) vs. the orthologous peptide in *D. mojavensis* ( *y* -axis).** Amino acid number is indicated along the left and bottom; coding-CDS number is indicated along the top and right, and CDSs are also highlighted with alternating colors. In *D. mojavensis* , the seventh coding CDS in *D. melanogaster* is split into two, with the location of the split denoted by a blue arrow in the dot plot. Regions which lack sequence similarity are highlighted in green and purple, labeled Box X and Y, respectively. **(D) Protein alignment of gig-PA in *D. melanogaster* and the orthologous peptide in *D. mojavensis.*** The symbols in the match line denote the level of similarity between the aligned residues. An asterisk (*) indicates that the aligned residues are identical. A colon (:) indicates the aligned residues have highly similar chemical properties—roughly equivalent to scoring > 0.5 in the Gonnet PAM 250 matrix (Gonnet et al., 1992). A period (.) indicates that the aligned residues have weakly similar chemically properties—roughly equivalent to scoring > 0 and ≤ 0.5 in the Gonnet PAM 250 matrix. A space indicates a gap or mismatch when the aligned residues have a complete lack of similarity—roughly equivalent to scoring ≤ 0 in the Gonnet PAM 250 matrix. The green and purple box labeled X and Y correspond to small regions of dissimilarity between the protein sequence of gig-PA in *D. melanogaster* with that of the putative ortholog.

## Description

**Table:** 

*This article reports a predicted gene model generated by undergraduate work using a structured gene model annotation protocol defined by the Genomics Education Partnership (GEP; thegep.org) for Course-based Undergraduate Research Experience (CURE). The following information in this box may be repeated in other articles submitted by participants using the same GEP CURE protocol for annotating Drosophila species orthologs of Drosophila melanogaster genes in the insulin signaling pathway.* "In this GEP CURE protocol students use web-based tools to manually annotate genes in non-model *Drosophila* species based on orthology to genes in the well-annotated model organism fruitfly *Drosophila melanogaster* . The GEP uses web-based tools to allow undergraduates to participate in course-based research by generating manual annotations of genes in non-model species (Rele et al., 2023). Computational-based gene predictions in any organism are often improved by careful manual annotation and curation, allowing for more accurate analyses of gene and genome evolution (Mudge and Harrow 2016; Tello-Ruiz et al., 2019). These models of orthologous genes across species, such as the one presented here, then provide a reliable basis for further evolutionary genomic analyses when made available to the scientific community.” (Myers et al., 2024). “The particular gene ortholog described here was characterized as part of a developing dataset to study the evolution of the Insulin/insulin-like growth factor signaling pathway (IIS) across the genus *Drosophila* . The Insulin/insulin-like growth factor signaling pathway (IIS) is a highly conserved signaling pathway in animals and is central to mediating organismal responses to nutrients (Hietakangas and Cohen 2009; Grewal 2009).” (Myers et al., 2024). “ *D.* *mojavensis* (NCBI:txid7230) is part of the *mulleri complex* in the *repleta* species group within the subgenus *Drosophila* of the genus *Drosophila* (Wasserman 1992; Durando et al., 2000) *.* It was first described by Patterson (Patterson and Crow 1940). *D. mojavensis* specializes on rotting cactus as its host and is found in the Mojave and Sonoran Deserts of the southwestern United States and northwestern Mexico including the Baja Peninsula, as well as on the channel-islands off the coast of California (https://www.taxodros.uzh.ch, accessed 1 Feb 2023).” (Congleton et al., 2023).

We propose a gene model for the *D. mojavensis* ortholog of the *D. melanogaster* *gigas* ( *gig* ) gene. The genomic region of the ortholog corresponds to the uncharacterized protein LOC6583279 (RefSeq accession XP_002008952.2 ) in the Dmoj_CAF1 Genome Assembly of *D. mojavensis* (GenBank Accession: GCA_000005175.1 , *Drosophila* 12 Genomes Consortium et al., 2007). This model is based on RNA-Seq data from *D. mojavensis* ( SRP006203 - Chen et al., 2014) and *gig* in *D. melanogaster* using FlyBase release FB2022_04 ( GCA_000001215.4 ; Larkin et al., 2021; Gramates et al., 2022; Jenkins et al., 2022).

Gene *gig* (short for gigas, aka *TSC2, CG6975, FBgn0005198* ) encodes a tumor suppressor protein that controls cell size, cell proliferation, and organ size (Ito and Rubin, 1999). The gig protein contains a GTPase-activating protein (GAP) domain and forms a complex with protein Tsc1 (Gao et al., 2001; Potter et al., 2001). The gig-Tsc1 protein complex promotes GTP hydrolysis of the small G-protein Rheb (Ras homolog enriched in brain), thereby antagonizing the insulin and TOR signaling pathways (Gao et al., 2002; Zhang et al., 2003). This gene was originally identified in humans as TSC2, and mutations in TSC1 or TSC2 result in tuberous sclerosis, a syndrome characterized by widespread benign tumors (European Consortium 1993; van Slegtenhorst et al., 1997).

***Synteny***

The referece gene, *gig* , occurs on chromosome 3L in *D. melanogaster* and nests two genes, *obstructor-J* ( *obst-J* ) and *CG7335 . gig* is flanked upstream by *CG14187* and *CG7365* and downstream by *CG7328* and *Snakeskin* ( *Ssk* ) that is nested by *CG42674* . The *tblastn* search of *D. melanogaster* gig-PA (query) against the *D. mojavensis* (GenBank Accession: GCA_000005175.1 ) Genome Assembly (database) placed the putative ortholog of *gig* within scaffold scaffold_6680 ( CH933809.1 ) at locus LOC6583279 ( XP_002008952.2 )— with an E-value of 0.0 and a percent identity of 57.97%. Furthermore, the putative ortholog nests two genes: LOC6583281 ( XP_002008954.1 ) and LOC6583280 ( XP_002008953.1 ) (E-value: 5e-58 and 4e-135; identity: 36.19% and 54.57%, respectively) which correspond to *obst-J* and *CG7335* in *D. melanogaster,* as determined by *blastp* ; Figure 1A, Altschul et al., 1990) *.* The putative ortholog is flanked upstream by LOC6583283 ( XP_002008956.1 ) and LOC6583282 ( XP_002008955.1 ) which correspond to *Grip163* ( *Grip163* ) and *endosulfine* ( *endos* ) in *D. melanogaster* (E-value: 0.0 and 9e-62; identity: 38.99% and 90.00%, respectively, as determined by *blastp* ). The putative ortholog of *gig* is flanked downstream by LOC6583278 ( XP_002008951.1 ) and LOC6583276 ( XP_002008949.1 ) that is nested by LOC6583274 ( XP_043865822.1 ); correspond to *CG7328* , *Ssk* and *CG42674* in *D. melanogaster* (E-value: 3e-146, 1e-112 and 0.0; identity: 64.06%, 96.30% and 79.40%, respectively, as determined by *blastp* ). The putative ortholog assignment for *gig* in *D. mojavensis* is supported by the following evidence: The genes surrounding the *gig* ortholog are orthologous to the genes at the same locus in *D. melanogaster* and local synteny is nearly completely conserved, aside from the upstream neighborhood, and is supported by results generated from *blastp* , so we conclude that LOC6583279 is the correct ortholog of *gig* in *D. mojavensis* ( Figure 1A ).

***Protein Model***

*gig* in *D. mojavensis* has two unique protein-coding isoforms (gig-PA and gig-PB; Figure 1B ). mRNA isoforms ( *gig-RA* and *gig-RB* ) contain fifteen CDSs. Relative to the ortholog in *D. melanogaster* , the RNA CDS number is not conserved. There appears to be a splitting of the seventh CDS in *D. mojavensis* (CDS 7_905_0), denoted by a green arrow in the dot plot ( Figure 1C ), with a new CDS being produced and increasing the number of *gig-RA* CDSs in *D. mojavensis* to sixteen. The sequence of gig-PA in *D. mojavensis* has 69.73% identity (E-value: 0.0) with the protein-coding isoform gig-PA in *D. melanogaster* , as determined by *blastp* ( Figure 1C ). Differences were found in small regions which lack sequence similarity, highlighted by the green and purple boxes (Box X and Y) in both Figure 1C and Figure 1D, and a difference in length of CDS 12_905_1 in *D. mojavensis* , compared to *D. melanogaster* , which may be explained by an indel of 47 amino acids in the protein alignment (Box X, Figure 1D ). Coordinates of this curated gene model (gig-PA, gig-PB) are stored by NCBI at GenBank/BankIt (accession **BK064558 , BK064559 )** . These data are also archived in the CaltechDATA repository (see “Extended Data” section below).

***Special characteristics of the protein model***

**CDS Split:** In *D. mojavensis* , the seventh CDS of *gig* in *D. melanogaster* (CDS 7_905_0) is split. This produces a new CDS and increases the total number of coding CDSs of the *gig* ortholog in *D. mojavensis* to sixteen. The location of this split is denoted by a blue arrow in the provided dot plot and protein alignment ( Figure 1C and Figure 1D ).

**Regions of Low Conservation:** Several small regions of dissimilarity exist between the sequence of gig-PA in *D. melanogaster* and *D. mojavensis* . The most significant regions with low sequence conservation are found in CDS 12_905_1 (CDS eleven in *D. melanogaster* and CDS twelve in *D. mojavensis* ), highlighted by Box X and Y in green and purple, respectively. Analysis of the protein alignment reveals these regions can be attributed to nonmatching amino acid residues and a longer sequence for this CDS in *D. mojavensis* (Box X and Y, Figure 1D ).

**Potential Indel:** The difference in length of CDS 12_905_1 in *D. melanogaster* and *D. mojavensis* may be explained by an indel of 47 amino acids. Box X in Figures 1C and 1D indicate the region of this split, which is most notable by the shift in the main line of the dot plot.

## Methods

Detailed methods including algorithms, database versions, and citations for the complete annotation process can be found in Rele et al. (2023). Briefly, students use the GEP instance of the UCSC Genome Browser v.435 (https://gander.wustl.edu; Kent WJ et al., 2002; Navarro Gonzalez et al., 2021) to examine the genomic neighborhood of their reference IIS gene in the *D. melanogaster* genome assembly (Aug. 2014; BDGP Release 6 + ISO1 MT/dm6). Students then retrieve the protein sequence for the *D. melanogaster* reference gene for a given isoform and run it using *tblastn* against their target *Drosophila* species genome assembly on the NCBI BLAST server (https://blast.ncbi.nlm.nih.gov/Blast.cgi; Altschul et al., 1990) to identify potential orthologs. To validate the potential ortholog, students compare the local genomic neighborhood of their potential ortholog with the genomic neighborhood of their reference gene in *D. melanogaster* . This local synteny analysis includes at minimum the two upstream and downstream genes relative to their putative ortholog. They also explore other sets of genomic evidence using multiple alignment tracks in the Genome Browser, including BLAT alignments of RefSeq Genes, Spaln alignment of *D. melanogaster* proteins, multiple gene prediction tracks (e.g., GeMoMa, Geneid, Augustus), and modENCODE RNA-Seq from the target species. Detailed explanation of how these lines of genomic evidenced are leveraged by students in gene model development are described in Rele et al. (2023). Genomic structure information (e.g., CDSs, intron-exon number and boundaries, number of isoforms) for the *D. melanogaster* reference gene is retrieved through the Gene Record Finder (https://gander.wustl.edu/~wilson/dmelgenerecord/index.html; Rele et al *.,* 2023). Approximate splice sites within the target gene are determined using *tblastn* using the CDSs from the *D. melanogaste* r reference gene. Coordinates of CDSs are then refined by examining aligned modENCODE RNA-Seq data, and by applying paradigms of molecular biology such as identifying canonical splice site sequences and ensuring the maintenance of an open reading frame across hypothesized splice sites. Students then confirm the biological validity of their target gene model using the Gene Model Checker (https://gander.wustl.edu/~wilson/dmelgenerecord/index.html; Rele et al., 2023), which compares the structure and translated sequence from their hypothesized target gene model against the *D. melanogaster* reference gene model. At least two independent models for a gene are generated by students under mentorship of their faculty course instructors. Those models are then reconciled by a third independent researcher mentored by the project leaders to produce the final model. Note: comparison of 5' and 3' UTR sequence information is not included in this GEP CURE protocol (Gruys et al., 2025).

## References

[R1] Altschul SF, Gish W, Miller W, Myers EW, Lipman DJ (1990). Basic local alignment search tool.. J Mol Biol.

[R2] Chen ZX, Sturgill D, Qu J, Jiang H, Park S, Boley N, Suzuki AM, Fletcher AR, Plachetzki DC, FitzGerald PC, Artieri CG, Atallah J, Barmina O, Brown JB, Blankenburg KP, Clough E, Dasgupta A, Gubbala S, Han Y, Jayaseelan JC, Kalra D, Kim YA, Kovar CL, Lee SL, Li M, Malley JD, Malone JH, Mathew T, Mattiuzzo NR, Munidasa M, Muzny DM, Ongeri F, Perales L, Przytycka TM, Pu LL, Robinson G, Thornton RL, Saada N, Scherer SE, Smith HE, Vinson C, Warner CB, Worley KC, Wu YQ, Zou X, Cherbas P, Kellis M, Eisen MB, Piano F, Kionte K, Fitch DH, Sternberg PW, Cutter AD, Duff MO, Hoskins RA, Graveley BR, Gibbs RA, Bickel PJ, Kopp A, Carninci P, Celniker SE, Oliver B, Richards S (2014). Comparative validation of the D. melanogaster modENCODE transcriptome annotation.. Genome Res.

[R3] Congleton H, Kiser CA, Colom Diaz PA, Schlichting E, Walton DA, Long LJ, Reed LK, Martinez-Cruzado JC, Rele CP (2022). Drosophila mojavensis - chico.. MicroPubl Biol.

[R4] Clark AG, Eisen MB, Smith DR, Bergman CM, Oliver B, Markow TA, Kaufman TC, Kellis M, Gelbart W, Iyer VN, Pollard DA, Sackton TB, Larracuente AM, Singh ND, Abad JP, Abt DN, Adryan B, Aguade M, Akashi H, Anderson WW, Aquadro CF, Ardell DH, Arguello R, Artieri CG, Barbash DA, Barker D, Barsanti P, Batterham P, Batzoglou S, Begun D, Bhutkar A, Blanco E, Bosak SA, Bradley RK, Brand AD, Brent MR, Brooks AN, Brown RH, Butlin RK, Caggese C, Calvi BR, Bernardo de Carvalho A, Caspi A, Castrezana S, Celniker SE, Chang JL, Chapple C, Chatterji S, Chinwalla A, Civetta A, Clifton SW, Comeron JM, Costello JC, Coyne JA, Daub J, David RG, Delcher AL, Delehaunty K, Do CB, Ebling H, Edwards K, Eickbush T, Evans JD, Filipski A, Findeiss S, Freyhult E, Fulton L, Fulton R, Garcia AC, Gardiner A, Garfield DA, Garvin BE, Gibson G, Gilbert D, Gnerre S, Godfrey J, Good R, Gotea V, Gravely B, Greenberg AJ, Griffiths-Jones S, Gross S, Guigo R, Gustafson EA, Haerty W, Hahn MW, Halligan DL, Halpern AL, Halter GM, Han MV, Heger A, Hillier L, Hinrichs AS, Holmes I, Hoskins RA, Hubisz MJ, Hultmark D, Huntley MA, Jaffe DB, Jagadeeshan S, Jeck WR, Johnson J, Jones CD, Jordan WC, Karpen GH, Kataoka E, Keightley PD, Kheradpour P, Kirkness EF, Koerich LB, Kristiansen K, Kudrna D, Kulathinal RJ, Kumar S, Kwok R, Lander E, Langley CH, Lapoint R, Lazzaro BP, Lee SJ, Levesque L, Li R, Lin CF, Lin MF, Lindblad-Toh K, Llopart A, Long M, Low L, Lozovsky E, Lu J, Luo M, Machado CA, Makalowski W, Marzo M, Matsuda M, Matzkin L, McAllister B, McBride CS, McKernan B, McKernan K, Mendez-Lago M, Minx P, Mollenhauer MU, Montooth K, Mount SM, Mu X, Myers E, Negre B, Newfeld S, Nielsen R, Noor MA, O'Grady P, Pachter L, Papaceit M, Parisi MJ, Parisi M, Parts L, Pedersen JS, Pesole G, Phillippy AM, Ponting CP, Pop M, Porcelli D, Powell JR, Prohaska S, Pruitt K, Puig M, Quesneville H, Ram KR, Rand D, Rasmussen MD, Reed LK, Reenan R, Reily A, Remington KA, Rieger TT, Ritchie MG, Robin C, Rogers YH, Rohde C, Rozas J, Rubenfield MJ, Ruiz A, Russo S, Salzberg SL, Sanchez-Gracia A, Saranga DJ, Sato H, Schaeffer SW, Schatz MC, Schlenke T, Schwartz R, Segarra C, Singh RS, Sirot L, Sirota M, Sisneros NB, Smith CD, Smith TF, Spieth J, Stage DE, Stark A, Stephan W, Strausberg RL, Strempel S, Sturgill D, Sutton G, Sutton GG, Tao W, Teichmann S, Tobari YN, Tomimura Y, Tsolas JM, Valente VL, Venter E, Venter JC, Vicario S, Vieira FG, Vilella AJ, Villasante A, Walenz B, Wang J, Wasserman M, Watts T, Wilson D, Wilson RK, Wing RA, Wolfner MF, Wong A, Wong GK, Wu CI, Wu G, Yamamoto D, Yang HP, Yang SP, Yorke JA, Yoshida K, Zdobnov E, Zhang P, Zhang Y, Zimin AV, Baldwin J, Abdouelleil A, Abdulkadir J, Abebe A, Abera B, Abreu J, Acer SC, Aftuck L, Alexander A, An P, Anderson E, Anderson S, Arachi H, Azer M, Bachantsang P, Barry A, Bayul T, Berlin A, Bessette D, Bloom T, Blye J, Boguslavskiy L, Bonnet C, Boukhgalter B, Bourzgui I, Brown A, Cahill P, Channer S, Cheshatsang Y, Chuda L, Citroen M, Collymore A, Cooke P, Costello M, D'Aco K, Daza R, De Haan G, DeGray S, DeMaso C, Dhargay N, Dooley K, Dooley E, Doricent M, Dorje P, Dorjee K, Dupes A, Elong R, Falk J, Farina A, Faro S, Ferguson D, Fisher S, Foley CD, Franke A, Friedrich D, Gadbois L, Gearin G, Gearin CR, Giannoukos G, Goode T, Graham J, Grandbois E, Grewal S, Gyaltsen K, Hafez N, Hagos B, Hall J, Henson C, Hollinger A, Honan T, Huard MD, Hughes L, Hurhula B, Husby ME, Kamat A, Kanga B, Kashin S, Khazanovich D, Kisner P, Lance K, Lara M, Lee W, Lennon N, Letendre F, LeVine R, Lipovsky A, Liu X, Liu J, Liu S, Lokyitsang T, Lokyitsang Y, Lubonja R, Lui A, MacDonald P, Magnisalis V, Maru K, Matthews C, McCusker W, McDonough S, Mehta T, Meldrim J, Meneus L, Mihai O, Mihalev A, Mihova T, Mittelman R, Mlenga V, Montmayeur A, Mulrain L, Navidi A, Naylor J, Negash T, Nguyen T, Nguyen N, Nicol R, Norbu C, Norbu N, Novod N, O'Neill B, Osman S, Markiewicz E, Oyono OL, Patti C, Phunkhang P, Pierre F, Priest M, Raghuraman S, Rege F, Reyes R, Rise C, Rogov P, Ross K, Ryan E, Settipalli S, Shea T, Sherpa N, Shi L, Shih D, Sparrow T, Spaulding J, Stalker J, Stange-Thomann N, Stavropoulos S, Stone C, Strader C, Tesfaye S, Thomson T, Thoulutsang Y, Thoulutsang D, Topham K, Topping I, Tsamla T, Vassiliev H, Vo A, Wangchuk T, Wangdi T, Weiand M, Wilkinson J, Wilson A, Yadav S, Young G, Yu Q, Zembek L, Zhong D, Zimmer A, Zwirko Z, Jaffe DB, Alvarez P, Brockman W, Butler J, Chin C, Gnerre S, Grabherr M, Kleber M, Mauceli E, MacCallum I, Drosophila 12 Genomes Consortium. (2007). Evolution of genes and genomes on the Drosophila phylogeny.. Nature.

[R5] Durando CM, Baker RH, Etges WJ, Heed WB, Wasserman M, DeSalle R (2000). Phylogenetic analysis of the repleta species group of the genus Drosophila using multiple sources of characters.. Mol Phylogenet Evol.

[R6] European Chromosome 16 Tuberous Sclerosis Consortium (1993). Identification and characterization of the tuberous sclerosis gene on chromosome 16.. Cell.

[R7] Gao X, Pan D (2001). TSC1 and TSC2 tumor suppressors antagonize insulin signaling in cell growth.. Genes Dev.

[R8] Gao X, Zhang Y, Arrazola P, Hino O, Kobayashi T, Yeung RS, Ru B, Pan D (2002). Tsc tumour suppressor proteins antagonize amino-acid-TOR signalling.. Nat Cell Biol.

[R9] Gonnet GH, Cohen MA, Benner SA (1992). Exhaustive matching of the entire protein sequence database.. Science.

[R10] Gramates L Sian, Agapite Julie, Attrill Helen, Calvi Brian R, Crosby Madeline A, dos Santos Gilberto, Goodman Joshua L, Goutte-Gattat Damien, Jenkins Victoria K, Kaufman Thomas, Larkin Aoife, Matthews Beverley B, Millburn Gillian, Strelets Victor B, Perrimon Norbert, Gelbart Susan Russo, Agapite Julie, Broll Kris, Crosby Lynn, dos Santos Gil, Falls Kathleen, Gramates L Sian, Jenkins Victoria, Longden Ian, Matthews Beverley, Seme Jolene, Tabone Christopher J, Zhou Pinglei, Zytkovicz Mark, Brown Nick, Antonazzo Giulia, Attrill Helen, Garapati Phani, Goutte-Gattat Damien, Larkin Aoife, Marygold Steven, McLachlan Alex, Millburn Gillian, Öztürk-Çolak Arzu, Pilgrim Clare, Trovisco Vitor, Calvi Brian, Kaufman Thomas, Goodman Josh, Krishna Pravija, Strelets Victor, Thurmond Jim, Cripps Richard, Lovato TyAnna, the FlyBase Consortium (2022). FlyBase: a guided tour of highlighted features. Genetics.

[R11] Grewal SS (2008). Insulin/TOR signaling in growth and homeostasis: a view from the fly world.. Int J Biochem Cell Biol.

[R12] Gruys ML, Sharp MA, Lill Z, Xiong C, Hark AT, Youngblom JJ, Rele CP, Reed LK (2025). Gene model for the ortholog of Glys in Drosophila simulans.. MicroPubl Biol.

[R13] Hietakangas V, Cohen SM (2009). Regulation of tissue growth through nutrient sensing.. Annu Rev Genet.

[R14] Ito N, Rubin GM (1999). gigas, a Drosophila homolog of tuberous sclerosis gene product-2, regulates the cell cycle.. Cell.

[R15] Jenkins VK, Larkin A, Thurmond J, FlyBase Consortium (2022). Using FlyBase: A Database of Drosophila Genes and Genetics.. Methods Mol Biol.

[R16] Kent WJ, Sugnet CW, Furey TS, Roskin KM, Pringle TH, Zahler AM, Haussler D (2002). The human genome browser at UCSC.. Genome Res.

[R17] Larkin A, Marygold SJ, Antonazzo G, Attrill H, Dos Santos G, Garapati PV, Goodman JL, Gramates LS, Millburn G, Strelets VB, Tabone CJ, Thurmond J, FlyBase Consortium. (2021). FlyBase: updates to the Drosophila melanogaster knowledge base.. Nucleic Acids Res.

[R18] Mudge JM, Harrow J (2016). The state of play in higher eukaryote gene annotation.. Nat Rev Genet.

[R19] Myers A, Hoffman A, Natysin M, Arsham AM, Stamm J, Thompson JS, Rele CP, Reed LK (2024). Gene model for the ortholog Myc in Drosophila ananassae.. MicroPubl Biol.

[R20] Navarro Gonzalez J, Zweig AS, Speir ML, Schmelter D, Rosenbloom KR, Raney BJ, Powell CC, Nassar LR, Maulding ND, Lee CM, Lee BT, Hinrichs AS, Fyfe AC, Fernandes JD, Diekhans M, Clawson H, Casper J, Benet-Pagès A, Barber GP, Haussler D, Kuhn RM, Haeussler M, Kent WJ (2021). The UCSC Genome Browser database: 2021 update.. Nucleic Acids Res.

[R21] Patterson JT and JF Crow, 1940. Hybridization in the mulleri group of Drosophila. *Univ. Texas Publs, 4032, 167-189*

[R22] Potter CJ, Huang H, Xu T (2001). Drosophila Tsc1 functions with Tsc2 to antagonize insulin signaling in regulating cell growth, cell proliferation, and organ size.. Cell.

[R23] Raney BJ, Dreszer TR, Barber GP, Clawson H, Fujita PA, Wang T, Nguyen N, Paten B, Zweig AS, Karolchik D, Kent WJ (2013). Track data hubs enable visualization of user-defined genome-wide annotations on the UCSC Genome Browser.. Bioinformatics.

[R24] Rele Chinmay P., Sandlin Katie M., Leung Wilson, Reed Laura K. (2023). Manual annotation of Drosophila genes: a Genomics Education Partnership protocol. F1000Research.

[R25] Tello-Ruiz MK, Marco CF, Hsu FM, Khangura RS, Qiao P, Sapkota S, Stitzer MC, Wasikowski R, Wu H, Zhan J, Chougule K, Barone LC, Ghiban C, Muna D, Olson AC, Wang L, Ware D, Micklos DA (2019). Double triage to identify poorly annotated genes in maize: The missing link in community curation.. PLoS One.

[R26] van Slegtenhorst M, de Hoogt R, Hermans C, Nellist M, Janssen B, Verhoef S, Lindhout D, van den Ouweland A, Halley D, Young J, Burley M, Jeremiah S, Woodward K, Nahmias J, Fox M, Ekong R, Osborne J, Wolfe J, Povey S, Snell RG, Cheadle JP, Jones AC, Tachataki M, Ravine D, Sampson JR, Reeve MP, Richardson P, Wilmer F, Munro C, Hawkins TL, Sepp T, Ali JB, Ward S, Green AJ, Yates JR, Kwiatkowska J, Henske EP, Short MP, Haines JH, Jozwiak S, Kwiatkowski DJ (1997). Identification of the tuberous sclerosis gene TSC1 on chromosome 9q34.. Science.

[R27] Wasserman, M. (1992). Cytological evolution of the Drosophila repleta species group. *Krimbas, Powell, 1992* : 455-552. FBrf0063954

[R28] Zhang Y, Gao X, Saucedo LJ, Ru B, Edgar BA, Pan D (2003). Rheb is a direct target of the tuberous sclerosis tumour suppressor proteins.. Nat Cell Biol.

